# *PPM1D/WIP1* in medulloblastoma

**DOI:** 10.18632/oncoscience.318

**Published:** 2016-09-03

**Authors:** Mwangala P. Akamandisa, Rita Nahta, Robert C. Castellino

**Affiliations:** Department of Pediatrics, Aflac Cancer and Blood Disorders Center, Children's Healthcare of Atlanta, Winship Cancer Institute, Emory University, Atlanta, GA 30322, USA

**Keywords:** medulloblastoma, WIP1, Hedgehog, PPM1D, CXCR4

Medulloblastoma is the most common malignant brain tumor in children. Current multimodal treatment regimens consisting of surgery, radiation, and chemotherapy, as well as advances in imaging, have contributed to increased survival rates over the past decade. Patients with newly diagnosed disease demonstrate survival rates that range from less than 50% to greater than 90%, depending on the molecular subgroup [1]. However, survivors can develop severe, long-term treatment-related neurologic, cognitive, and endocrine sequelae. In addition, approximately 30% of patients experience disease recurrence, for which there are currently no effective standard therapies. The median survival of patients with relapsed/refractory medulloblastoma is less than one year, indicating a major unmet clinical need.

Development of effective therapies for relapsed/refractory medulloblastomas requires an understanding of the mechanisms mediating disease progression and/or drug resistance. Gene expression profiling studies have demonstrated that medulloblastomas can be stratified into at least four molecularly-distinct subgroups according to activation of *wingless* (WNT) or *sonic hedgehog* (SHH) signaling, or as Group 3 or Group 4 tumors. Medulloblastomas with nodular, desmoplastic histology usually exhibit active *SHH* signaling and are generally associated with an excellent survival prognosis. One study reported that high expression of the G protein- coupled receptor, CXCR4, defines a distinct sub- group of *SHH*-driven medulloblastomas that are more common in younger patients and characterized by a desmoplastic histology [2]. However, in the presence of *GLI2* amplification or *TP53* mutation, *SHH*-activated medulloblastomas demonstrate a dismal survival prognosis of less than 50% [1]. Patients with Group 3 medulloblastoma have an increased incidence of poor prognostic features, including *MYC* amplification, gain of chromosome 17q, and leptomeningeal metastasis at diagnosis, along with the worst prognosis for survival among all medulloblastoma subgroups. Group 4 tumors constitute the largest medulloblastoma subgroup and also exhibit gain of chromosome 17q, with or without 17p loss, along with leptomeningeal metastasis. However, survival is intermediate between that of *WNT* and Group 3 medulloblastomas.

*PPM1D/WIP1* (*protein phosphatase, magnesium- dependent 1, delta; wild-type TP53-induced phosphatase1*) on chromosome 17q22-23 encodes a serine/threonine protein phosphatase, which regulates DNA damage response, in part through inhibition of p53. Amplification, overexpression, or mutation in the C-terminal domain causes WIP1 to gain oncogenic functions. Approximately two-thirds of medulloblastomas demonstrate amplification or overexpression of *WIP1* [3, 4]. We previously demonstrated that patients with medulloblastomas that express high levels of *WIP1* have an increased incidence of metastasis and inferior progression-free and overall survival [5]. Gene expression analysis indicated up-regulation of metastasis genes, including *matrix metalloproteinase 9* (*MMP9*) and *CXCR4*, in medulloblastoma cell lines with high WIP1 expression. Increased CXCR4 expression led to Akt activation and increased growth and invasion of *WIP1* over-expressing medulloblastoma cells under cell culture conditions and as orthotopic cerebellar xenografts. WIP1 did not control transcription of CXCR4, but promoted re-localization of CXCR4 to the medulloblastoma cell membrane by suppressing expression of *G protein receptor kinase 5* (*GRK5*) and inhibiting phosphorylation of the GRK5 target, serine 339, on CXCR4.

More recently, we have also shown that *WIP1* promotes medulloblastoma formation in the context of *SHH* pathway activation. *WIP1* overexpression increased expression of the *SHH* target genes *Gli1* and *Ccnd1*, and increased proliferation in response to SHH stimulation in cerebellar granule neuron precursor cells, a validated medulloblastoma cell of origin, in a p53-independent manner *in vitro* as well as in the developing cerebellum of *WIP1* transgenic mice [6]. Cross-talk between *WIP1* and *SHH* signaling was further supported by our discovery that *WIP1* transgenic mice, when crossed with *SHH*-activated mice, exhibited an increased incidence of medulloblastoma and reduced survival. Conversely, knock-out of the *Wip1* gene significantly suppressed medulloblastoma formation in *SHH*-activated mouse models. And, treatment with a small molecule WIP1 inhibitor, suppressed the growth of short-term cultures of medulloblastoma cells derived from *Shh*-activated mice.

The discovery of unique signaling networks involving *WIP1, CXCR4*, and *GRK5* and cross-talk between the *SHH* signaling pathway and *WIP1* provide potential insights into mechanisms through which WIP1 promotes medulloblastoma tumor growth and metastasis. *WIP1* knockout blocked medulloblastoma formation in *SHH*-activated mice [6], and pharmacologic inhibition of WIP1 reduced *in vivo* growth of medulloblastoma cell lines [7]. Dual inhibition of WIP1 and SHH signaling yielded increased growth inhibitory effects in *SmoA1*/*SmoA1* and tamoxifen-induced *Math1*-creER; *Ptc1 fl/fl* mice [6]. Treatment with the CXCR4 antagonist AMD3100 has also been shown to reduce intracranial tumor growth in a medulloblastoma xenograft mouse model [2]. These results provide rationale for targeting WIP1 alone or in combination with drugs that target SHH or CXCR4 pathways in relapsed/refractory medulloblastoma. Future studies should examine dual inhibition of WIP1 and CXCR4 or WIP1 and SHH in pre-clinical models and in the setting of relapsed/refractory medulloblastoma.
